# Pre-competition habits and injuries in Taekwondo athletes

**DOI:** 10.1186/1471-2474-6-26

**Published:** 2005-05-27

**Authors:** Mohsen Kazemi, Heather Shearer, Young Su Choung

**Affiliations:** 1 Clinical education, Canadian Memorial Chiropractic College, Toronto, Ontario, Canada; 2 Clinical Sciences Resident, Canadian Memorial Chiropractic College, Toronto, Ontario, Canada; 3 former Canadian Taekwondo Team Head Coach, current Ontario Taekwondo Association President, Toronto, Ontario, Canada

## Abstract

**Background:**

Over the past decade, there has been heightened interest in injury rates sustained by martial arts athletes, and more specifically, Taekwondo athletes. Despite this interest, there is a paucity of research on pre-competition habits and training of these athletes. The purpose of this pilot study was to assess training characteristics, competition preparation habits, and injury profiles of Taekwondo athletes.

**Methods:**

A retrospective survey of Canadian male and female Taekwondo athletes competing in a national tournament was conducted. Competitors at a Canadian national level tournament were given a comprehensive survey prior to competition. Items on training characteristics, diet, and injuries sustained during training and competition were included. Questionnaires were distributed to 60 athletes.

**Results:**

A response rate of 46.7% was achieved. Of those that responded, 54% dieted prior to competition, and 36% dieted and exercised pre-competition. Sixty-four percent of the athletes practised between 4–6 times per week, with 54% practicing 2 hours per session. Lower limb injuries were the most common (46.5%), followed by upper extremity (18%), back (10%), and head (3.6%). The majority of injuries consisted of sprains/strains (45%), followed by contusions, fractures, and concussions. More injuries occurred during training, including 59% of first injuries.

**Conclusion:**

More research needs to be conducted to further illustrate the need for appropriate regulations on weight cycling and injury prevention.

## Background

The martial arts have their beginnings in the Orient but more specifically the common styles seen in Western society are from Japan, China, and Korea. Taekwondo, which originated in Korea more than 1000 years ago, is more sport than self-defense oriented [1]. In 2000, Taekwondo became recognized as an official sport at the Sydney Olympics. Taekwondo is a full contact free-sparring sport which awards points for head contact. As such, there has been increased interest in injury rates in the sport, especially relating to head injuries [2-5]. Although much of the research focuses on injury rates, very little examination into pre-competition habits and training has been conducted. The current authors felt that certain key areas needed to be addressed. These included training habits, injuries, dietary practices, and social support.

### Training habits and injury

Most martial arts athletes practice between two to four times per week [1]. However, like in any sport, the frequency and hours of martial arts training can vary widely depending on athletic and competitive level of the individual. Training may be defined as a routine or process undertaken by athletes to further enhance their skill. Specific training may vary among each athlete, but there is usually a general format which is followed. Training classes often begin with a brief warm-up or stretching routine. This may then be followed by kicking drills, self-defense drills, training in patterns (forms) and sparring [1]. Taekwondo athletes have a wide variety of protective equipment available, although its use varies greatly, and concerns have been raised that the equipment often protects the attacker more than the defender [2]. Besides the regular training routine, students also participate in full-contact tournaments.

At the time of data collection at the tournament in question, one point was awarded for any strike from the waist upward. The World Taekwondo Federation (WTF) rules and regulations for Olympic competition were used for this tournament [7]. For the purpose of this study, an athlete was considered injured if the following occurred: 1) any circumstance which forced the athlete to leave the competition or training session; 2) any circumstance for which the referee or athlete had to stop the competition; or 3) any circumstance for which the athlete requested medical attention [6]. Injuries occur in both training and competition, and trauma to the lower extremity and head are the most common sites reported [6]. A distinction can be made between overuse and traumatic forms of injury; although in reality they could be considered as points along one continuum. Overuse injuries may occur following continued or accumulated microtrauma to a structure or body area [8]. Traumatic injuries are the result of physical trauma or external force to a certain region leading to a diminished functional ability [8]. In the current pilot study no distinction was made between these forms of injury although it would be an interesting feature to examine in future research. The majority of research about Taekwondo injuries has examined injuries sustained during competition. Even so, it has been noted that up to 60% of injuries are not reported [2]. There are a variety of explanations for this, including poor recall, lack of importance placed on the injury, and unwillingness to disappoint trainers.

### Weight cycling

Weight cycling is a term used to describe rapid weight loss following self-induced food limitation and/or dehydration. Both gradual (seasonal) and rapid (weekly) weight reduction cycles are used by athletes, and have been investigated for potential effects on nutrition and performance [9]. These cycles are used in various sports such as judo, rowing, wrestling, and boxing in order to make a weight category. Like many of these sports, Taekwondo consists of repeated-effort, high intensity physical demands. In addition to this, Taekwondo competition is structured in a similar fashion to boxing and rowing in that athletes are required to meet weight requirements in order to compete. Although there is no known reported research about weight cycling in Taekwondo, it is the primary author's experience (holding a fifth degree black belt in WTF Taekwondo and practicing Taekwondo for more than twenty-five years) that it is widely practiced in the sport. The WTF has various weight classes depending on competition level [7]. Table 1 illustrates the four WTF weight classes per gender for Olympic competition, which were also the categories used at the tournament for the present study. Although the World Taekwondo Federation has eight distinct weight classes per gender for all competitions and championships except for the Olympic Games, no rulings have been implemented to address weight cycling in the sport.

**Table 1:** World Taekwondo Federation Olympic Weight Classes for Men and Women

**Males**	**Females**
Less than 58 kg	Less than 49 kg
Between 58 to 68 kg	Between 49 to 57 kg
Between 68 to 80 kg	Between 57 to 67 kg
Over 80 kg	Over 67 kg

To date there has been no research investigating the perceived benefit of weight cycling among Taekwondo athletes and this is an area in which much work should be undertaken. Due to the similarities between boxing and Taekwondo, with respect to competition weigh-ins, it may be possible to infer that Taekwondo athlete's perceptions of this technique may be similar. One study examining weight cycling among boxers reported that all the subjects felt it necessary to lose weight prior to competition and that it improved their performance [10]. Athletes using this weight control technique may be mistaken in thinking that an advantage will be gained over the opponent competing at his/her natural weight. To this point the research findings into the effect of food and fluid restriction has been equivocal [10]. There is also a belief that nutrients and strength can be restored by eating and drinking in the period between the weigh-in and the competition. Several authors have reported various techniques for rapid weight loss. A few strategies include dieting, restricting food and fluid intake, diuretic use, long runs, skipping, cycling, saunas, and exercising in rubber/plastic suits [10-12].

### Psychological state / support

Despite athletes' perceptions of the benefits of weight cycling, there are both physiological and psychological side effects. Using the Profile of Mood States-A (POMS-A), anger, confusion, depression, fatigue, tension, and vigor were measured among weight cycling amateur boxers [10]. The study reported that rapid weight loss was associated with significantly higher scores on anger, fatigue, and tension, with decreased vigor. The authors concluded that weight cycling resulted in negative mood and debilitated performance among their respondents. It is important for parents, coaches, and significant others to recognize these signs and address them appropriately.

The idea of participating in competitive situations can be daunting for some individuals. Intense pressure, anxiety, and somatic manifestations may result. For those in athletic competition, it is vital to recognize and address these sources of stress in order to produce more successful outcomes.

To date there is a lack of research in the areas of weight cycling and its perceived benefits among Taekwondo athletes. There is also limited research in the areas of social support and injury profiles in Taekwondo athletes outside of competition. This pilot study is an initial step towards increasing our knowledge in these areas. The purpose of the present study was to assess training characteristics, competition preparation habits and injury profiles of Taekwondo athletes.

## Methods

### Subjects

Sixty Canadian male and female respondents were recruited for the study. Participants were Taekwondo athletes competing at a national-level tournament. A total of 28 respondents with an age range of 16 to 29 years returned the distributed questionnaire. Four females and 18 males completed the questionnaire. An additional six participants did not indicate their gender on the returned questionnaires. The mean age of the competitors was just over 22 years, with a mean height and weight of 68.6 inches and 148 pounds, respectively.

### Instrumentation

A twenty-one-item questionnaire (See Additional file: 1 was used to obtain a general profile of the athletes. Areas of focus included: amount of Taekwondo practice; training satisfaction; protective gear used; pre-competition eating habits; competition preparedness; social support for the sport; and injury profiles. The questionnaire was modelled after one that had been developed for Dragon Boat racers. Neither questionnaire has been tested for validity or reliability.

### Procedure

The first author was working at the national carding tournament as a member of the health care team. As they entered the facility, potential participants were invited to fill out the survey by the current author and his assistants. Only card-carrying athletes competing in the tournament were given questionnaires. Prior to participation, informed consent was obtained by the participants or their guardians. At that time, any questions regarding the study or survey were addressed. Sixty questionnaires were distributed. When participants were given the questionnaire, they were asked to complete it immediately and then return it to the current author or assistants.

### Statistics

The Statistica Release 6 statistical package was used for all analysis. Descriptive statistics and Pearson's chi-square test were used. When inputting data, it was noted that certain variables had missing responses. In these instances, the number of participants who completed the questions was used to calculate the results.

## Results

### Training Habits

Training time, measured by number of practices per week, and number of hours per training session, is outlined in Tables 2 and 3. Specific activities during training were also examined. The frequency of sparring was reported. Twenty-five percent (n = 7) of the competitors reported sparring one to two times per week. Over 53% (n = 15) of respondents sparred three to four times per week, and over 21% (n = 6) sparred five or more times per week. Pre- and post-training stretching was also reported. Over 40% (n = 11) of respondents reported only stretching prior to their training sessions, and close to 60% (n = 16) of respondents stretched both before and after training. When examining the use of warm-up and cool-down exercises, over 57% (n = 16) of participants noted always warming up prior to training, while almost 43% (n = 12) reported only warming up occasionally. Only six of 28 respondents (21%) reported they always engaged in post-training cool-down exercises, other than stretching. Over sixty-four and fourteen percent (n = 22) of respondents reported occasionally and never using cool down exercises, respectively.

**Table 2:** Training Time – Number of practices per week (n = 28)

**Practices Per Week**	
**Number of Practices**	**Percent**

2	7.1
3	14.3
4	25
5–6	39.3
7 or more	14.3

**Table 3:** Training Time – Number of hours per practice (n = 28)

**Hours Per Practice**	
**Number of Hours**	**Percent**

1	17.9
2	53.6
3	17.9
4	7.1
5 or more	3.6

The use of protective gear was also examined. Over 60 percent (n = 17) of respondents reported always using protective gear, while 39.3% (n = 11) only used it occasionally. Table 4 lists the type of gear and its percentage use by the respondents. When examining the frequency of missed practices, five respondents reported never having missed a practice even though they were injured. More athletes missed one to two practices when injured, although there were a few respondents who missed a substantial number of practices regardless of the injury frequency. Less time and practices were missed as injury number increased. Eight respondents reported that they had not experienced any injuries. Four cases had incomplete data and were subsequently not used in the calculation of missed practices.

**Table 4:** Type and frequency of protective gear used by Taekwondo athletes (n = 28)

**Gear Used**	Elbow Pads	Shoes	Shin Pads	Gloves	Head Gear	Instep Pads	Chest Protector	Mouth Guard
**% of Use**	57.1	35.7	92.9	3.6	57.1	10.7	78.6	14.3

Table 5 reports the number of missed practices for the first to fifth injuries.

**Table 5:** Number of missed practices affected by injury frequency (n = 16)

**# of Missed Practices**	**1^st ^Injury (n)**	**2^nd ^Injury (n)**	**3^rd ^Injury (n)**	**4^th ^Injury (n)**	**5^th ^Injury (n)**
None	3	0	1	0	1
1 to 2	5	1	0	1	0
3	1	0	0	0	0
5 to 9	1	1	0	0	0
10 to 14	1	2	0	0	0
20 to 24	2	0	0	0	0
25 to 29	0	0	1	1	0
30 to 39	1	0	0	0	0
40 to 49	1	1	0	0	0
50 to 99	1	0	0	0	0
150+	0	1	0	0	0

### Injury profile

At this level of competition, 75% (n = 21) had six or more years of Taekwondo experience, with over 57% (n = 16) of individuals having eight or more years experience. Of the 28 participants, only 6 (21%) reported never having experienced an injury. At a value of 46.5% (n = 13), the lower limb was reported as being the region most injured in first-time injuries. The upper limb and back had an injury rate of 18% (n = 5) and 10.8% (n = 3), respectively. Over 3% (n = 1) of participants reported experiencing head injuries. Data for participants suffering injuries are reported in Table 6. Seventy nine percent (n = 22) of athletes reported they had an injury. Of those, 47 percent (n = 13) had a lower limb injury, 18 percent (n = 5) had an upper limb injury, and 11 percent (n = 3) had a back injury. Thirteen athletes (46%) reported they had experienced a second injury. Of those, 29 percent (n = 8) had a lower limb injury, 11 percent (n = 3) had an upper limb injury, and seven percent (n = 2) had a back injury. Injury rates decreased substantially for those who reported experiencing a third injury. Only fourteen percent (n = 4) of the athletes reported suffering from a third injury. Of those, seven percent (n = 2) had a lower limb injury, while upper limb (n = 1) and back injuries (n = 1) each accounted for four percent of the injuries. Four athletes (14%) reported experiencing a fourth injury while 2 athletes (7%) reported they had a fifth injury. Of those, only lower limb injuries were reported.

**Table 6:** Injury rates and location of injuries in Taekwondo athletes (n = 24)

**Number of Injury**	**Lower Limb Injury (%)**	**Upper Limb Injury (%)**	**Back Injury (%)**	**Other Injury (%)**
1^st^	13 (46.5)	5 (17.9)	3 (10.8)	1 (3.6)
2^nd^	8 (28.5)	3 (10.8)	2 (7.2)	0
3^rd^	2 (7.2)	1 (3.6)	1 (3.6)	0
4^th^	4 (14.4)	0	0	0
5^th^	2 (7.2)	0	0	0

The frequency of injuries in training versus competition was also examined. Out of a total 22 responses, 13 respondents reported experiencing their first injury during training, while nine respondents experienced their first injury in competition. Training was most frequently reported as the time of injury, with eight out of thirteen respondents reporting second injuries occurring during training, versus five out of thirteen during competition. No competition injuries were reported for the third to fifth injuries.

In order to better understand the injury data, injury rates were calculated using the basic rate formula: (#injuries / # athlete-exposures) × 1000 = # injuries per 1000 athlete-exposures (A-E). Due to problematic data, only 24 of the 28 respondent's data were used. The overall rate of injuries was 520/1000 A-E. The injury rate was 354.2/1000 A-E for training, and 166.7/1000 A-E for competition. The injury rate for training per hour was 32.5/1000 A-E/ hour.

A variety of care was sought by the athletes following injury. Of those respondents completing the survey, 25% (n = 7) did not seek any form of treatment. Another 10.7% (n = 3) of the athletes were treated by medical doctors, 10.7% (n = 3) were treated by physiotherapist, 10.7% (n = 3) were treated by chiropractors, and 3.6% (n = 1) received acupuncture. A variety of treatment combinations were also reported by 14.4% (n = 4) of the athletes. These combinations included chiropractic care as well as various sources of therapy listed above.

### Weight cycling

Pre-competition habits are an important factor to examine in all sports. Due to weight classifications in Taekwondo, the athletes are very conscious of their weight. Certain trends were reported by participants in order to achieve the desired weight. Over 53% (n = 15) of participants reported fasting prior to the competition. Of these individuals, 33.3% (n = 4) neither ate nor drank, 50% (n = 6) only drank, and 17% (n = 2) ate but did not drink. Aerobic exercise was another method used by competitors in order to reach the desired weight category. In addition to dieting, 83%, or 10 of the 15 fasting participants reported doing aerobic activity prior to competition.

### Social support

Support is often key to athletes at higher levels of competition. The current author examined athlete support for the sport by significant others. Seventy-eight percent (n = 22) of athletes reported they had parental support, while 14% (n = 4) reported no parental support was given, and 7% (n = 2) of respondents responded that this support did not apply. Spousal or partner support was reported by 32% (n = 9) of the athletes, while 18% (n = 5) did not receive this support, and 50% (n = 14) of respondents noted that the category did not apply to them. Because the survey was completed prior to competing, participants were asked to record if they felt prepared for the upcoming event. Fifty percent (n = 14) of respondents responded that they were prepared, 39% (n = 11) felt prepared but nervous, and 11% (n = 3) did not feel adequately prepared for the competition.

Several comparison analyses were performed using Pearson's chi-square test. None of the values were of statistical significance, and thus not reported. The rationale for only reporting frequencies is due to the small sample size of the study, making the use of other analyses like Pearson's or Fisher's Exact Test erroneous.

## Discussion

The objective of this retrospective investigation was to assess training characteristics, competition preparation habits, and injury profiles of taekwondo athletes. By having the athletes complete a survey, several areas of concern regarding competition preparation and injuries were highlighted.

### Training and injuries

When examining the training habits of taekwondo athletes, the current study reviewed several components of performance. Respondents had significant experience in the sport, with over 75% having six or more years of involvement. Training time, measured by number of practices per week and number of hours per practice, was also high. Over 53% of practices were two hours, with over 45% of athletes practicing between two to four times per week. Of importance is the relationship of how training time and competition is affected by injury. Unfortunately, our sample size was too small to have meaningful comparisons.

Other authors have reviewed this relationship. In a study by Feehan and Waller [2], competitive performance affected by previous injury was examined. On the day of the competition, 35% of respondents had a current injury affecting performance. Some of these required strapping or support in order to perform. Seventeen percent reported continuing to train/compete against medical advice. Even with these injury rates, the authors noted that fight outcome was not significantly associated with current or previous injuries. One conclusion which might be drawn is that the injuries reported were not severe enough to negatively impact the athletes' performance. It can also be assumed that many of those with severe injuries would have chosen to withdraw from or not enter the competition until an appropriate level of health was reached.

Practice activities among the respondents varied. A large proportion of respondents warmed up prior to kicking drills, while less than 25% cooled down. One possible explanation for warm-up participation may be that it is encompassed within the class. On the other hand, cool downs may be left to the discretion of the athlete once training is finished. In the current study, stretching was considered a separate activity from warm-ups and cool-downs. Over 60% of respondents stretched both before and after training, while just over 40% stretched prior to training only. In future studies, it would be interesting to note if stretching occurred after warm-up, which is a newer trend of thinking in the prevention of muscle injury [13]. Within the questionnaire, it was specified that cool down exercises did not include stretching. By doing this, the authors intended to eliminate the overlap between the stretching and cool down items. Future studies should allow subject to specify the various types of cool down activities used, such as light jogging or light-paced jumping jacks. Future studies should also examine the relationship between injury rates and the use of stretching, warm-ups, and cool downs. Due to a limited sample size the current study was not able to make these comparisons. The final training activity examined in the current study was sparring. This was an integral part of taekwondo training, with over 50% of respondents sparring three to four times per week. The current study attempted to examine the relationship between frequencies of sparring when injured, but there were no statistically significant differences. Birrer [14] reported that most injuries occur during sparring, thus it is an area of training which deserves specific focus. Future studies should focus on both injury type and frequency occurring during sparring, as well as limitations in sparring due to injury.

In the current study, training was most frequently reported as the time of injury and relatively few injuries occurred during competition. Even so, the overall reported injury rate was quite high, at 520/1000 A-E. The injury rates calculated for both training and competition are likely to be skewed. Firstly, respondents were asked to simply report if they had been injured during competition or training. A more accurate representation may have occurred if athletes were asked to report how many competitions they had participated in during the previous year and if they had suffered injuries during any of these. Training injury rates may have also been affected by athletes returning to play prior to complete resolution of their problem. This could make athletes more susceptible to subsequent injuries.

When reviewing injury location reported in the current study, it was not surprising, that the lower extremity received the most injuries. This was also true for all subsequent injuries reported (up to five per athlete). These results are consistent with those of several other studies [2,5]. The upper limb was the second most frequently injured region, with the head being the least frequently injured. Sprains and strains were the most common injuries, followed by contusions, which is similar to other research [2]. Other reports have listed contusions and concussions as the most common forms of taekwondo-related injuries [3,6]. Zemper et al. [5] reported that contusions were the predominant type of injury in his study of injury rates recorded during the 1988 US Olympic team trials for taekwondo.

In general, the number of practices missed decreased with subsequent injuries. Also, there were relatively few athletes who missed a large number of practices. This is perhaps attributable to injury severity. One explanation for fewer missed practices could be that once an athlete experienced one injury, s/he was more likely to increase the use of protective gear, thus avoiding or decreasing future injury severity. Future studies should examine if there is a relationship between increased uses of protective gear following an injury.

Recently, there has been increased concern regarding head injuries in taekwondo. Koh and Watkinson [4] reported that when compared to other contact sports, competition Taekwondo had the highest incidence rate of concussions. This might be explained by the fact that athletes are awarded points for head contact. Disturbingly, it was also found that over 30% of concussed athletes suffered more than one significant head blow in the same match. Also, among 99% of the head blows, no evasive manoeuvres were attempted. This would suggest that athletes are poorly trained in blocking skills. Pieter and Zemper [3] also reported that contusions and cerebral concussions were the leading injury types among young male and female Taekwondo athletes. Again, unblocked attacks were a frequent occurrence in these injuries. Widespread safety education on head injuries, and more specifically concussions, is needed among Taekwondo athletes, trainers, and referees. Improved blocking skills and headgear are a priority in order to help avoid serious injury.

Following injury, a variety of care was sought by the current study's athletes. Interestingly, one quarter of the athletes chose not to seek any form of treatment. This could perhaps be accounted for by the athlete's perception of the injury being relatively minor, or being able to manage it without medical advice. Several health professional were consulted by the injured athletes. These included medical doctors, physiotherapist, chiropractors, acupuncturists, and massage therapists. In addition, several athletes consulted multiple therapists. Athletes are generally anxious to return to their pre-injury status, and often become impatient with long-term therapy. This may explain why multiple health professionals were consulted. Also, some health professionals realize the benefit to a multidisciplinary approach, and use a network of referral sources when necessary.

### Weight cycling

Not surprisingly, more than half of the competitors in the current study dieted prior to competition in order to make their weight class. Although the questionnaire did not specifically define fasting, the subsequent question provided several categories of fasting, such as "did not eat and drink", "did not drink but eat", and so on. Even with the lack of a clear definition for fasting, fifty percent of the participants reported having completely restricted food intake, while 33% fully restricted food and liquids. Because of the nature of this tournament setting, it was not feasible to weigh athletes prior to their competition. As such, the authors were not able to report actual weight loss occurrence among the athletes. Future studies should focus on intended and actual weight loss among Taekwondo athletes in order to better capture the occurrence of weight cycling in the sport.

Rapid weight loss is a common practice among athletes in weight class sports. Hall and Lane [10] reported that their boxing subjects lost an average of 5.16% of their body weight within one week. Along with the weight loss, subjects reported higher anger, fatigue, and tension, as well as reduced vigour. Participants were able to maintain their baseline performance of circuit training when at the reduced weight, although the scores were significantly lower than the athletes expected. It can be postulated that athletes have a misplaced sense of improved strength and performance capabilities when weight cycling for competition. Unfortunately, these views may be reinforced if a weight cycling athlete wins a competition, thus increasing the likelihood of using the strategies in the future. In a study by Alderman et al. [11] examining the prevalence of and weight loss techniques used by high school wrestlers, more successful wrestlers engaged in rapid weight loss (RWL) versus less successful wrestlers. This further reinforces the use of RWL among young competitors.

What is particularly striking are the methods used to induce rapid weight loss. Among high school wrestlers, excessive running was used by almost 92% of individuals practicing RWL. Exercising in rubber/plastic suits and using saunas are prohibited in American high school wrestling, but they continued to be used by 40–60% of wrestlers to achieve RWL [11]. Thirty-six percent of respondents in the current study did aerobic exercise in addition to dieting to make their weight, but specific activities were not asked for in the survey.

Many short term and long term side effects have been reported with rapid weight loss. Alderman et al. [11] reported multiple symptoms experienced by collegiate weight cycling wrestlers. Over 46% of participants experienced headaches, while over 44% and 42% experienced dizziness and nausea, respectively. Other symptoms included hot flashes, nosebleeds, feverish sensations, disorientation, and increased heart rate. Wenos and Amato [15] reported that college-level wrestlers also experienced an increased perception of effort as muscle strength and endurance decreased with rapid weight loss.

Fogelholm et al. [9] studied the effects of gradual versus rapid weight loss in national wrestlers and judo athletes on nutrient intake, micronutrient status, and physical performance (sprint, jump height, and anaerobic performance). A 5% to 6% reduction in body weight was reported in the gradual and rapid loss groups. Nutrient intake was significantly decreased in both groups in B1, B2, K, Ca, Mg, Fe, and Zn values, compared to baseline measures. Speed, vertical jump, and anaerobic performance were not impaired by either rapid or gradual weight loss. Other studies have also reported that despite nutrient depletion, performance of Olympic level amateur boxers during rapid weight loss was not significantly different versus times of normal dietary behavior. These authors concluded that despite reduced carbohydrate intake, there were other sufficient energy sources to meet performance demands [16]. In contrast, Filare et al. [12] reported that all mean micronutrient intakes were below recommended values, while triglyceride levels and free fatty acids were increased in weight cycling judo athletes. Left hand grip values and 30-second jump test output were decreased after seven days of food restriction.

By reviewing the literature, some might argue that the evidence of health risks from weight cycling is equivocal. Even so, there are several possibilities that may help explain the lack of supporting data. One possibility is that there may be no effect. Another proposed by Waslen, McCargar, and Taunton [17], is that the duration, frequency, and severity of food restriction among the judo athletes in their study may not have been sufficient to have an effect. Even with a lack of strong support to illustrate the ill effects of weight cycling, monitoring dietary habits of athletes in weight class sports is recommended. It is more prudent to assume that larger weight losses and more frequent dieting could potentially result in negative physiological and performance consequences. Widespread regulations need to be implemented to control weight cycling practices among weight class sports. Athletes need to be educated regarding the negative effects of the practice on both their health and performance.

### Psychological state/support

Support is often key to athletes at higher levels of competition. The current authors examined athlete support by significant others. The majority of athletes reported receiving support from either their parents, or spouse/partner. Unfortunately, the questionnaire used in this study did not delve into the various aspects of psychological state or support. In this pilot study, respondents were simply asked "Are your parents supportive of your involvement in Taekwondo?" and "Is your spouse or significant other supportive of your involvement in Taekwondo?" It is obvious that neither of these questions addresses the various components involved in support. Future studies need to be more specific in questioning the types and level of support provided to athletes, whether it be emotional, financial, or other various forms of support. As such, these results are of little contributive value. It should be noted that although a large percentage of the athletes felt prepared for the competition, they also reported being nervous. The significance of anxiety and other personality traits in competitive sport has long been studied. It has been reported that winning Taekwondo athletes had lower cognitive and somatic anxiety and higher self-confidence then their losing counterparts [18]. Others found no support for the relationship between competition trait anxiety and Taekwondo performance [19]. Even so, for ultimate personal success, athletes often require a strong support base. This encompasses a sense of understanding, trust, and support from the trainer, and significant others.

In weight class sports, the potential effects of weight cycling must also be kept in mind. As noted above, several studies have reported deleterious effects associated with rapid weight loss. These effects may involve one's mental status. Filaire et al. [12] reported that confusion, anger, fatigue, and tension were significantly higher after weight loss. Vigor was also significantly lower after food restriction. Thus, when considering the psychological preparedness of an athlete, multiple factors must be measured.

There are a few limitations in the present study which need to be addressed. The most obvious methodological issue in this study is that the questionnaire has not been validated. There is very little reported research regarding precompetition habits among Taekwondo athletes. As such, the authors felt it necessary to develop the questionnaire, knowing that there would be issues with its validity. Because this is a pilot study, the results from this study should be used with caution and as a means to enhance future studies in this area. In addition, the small sample size significantly affected statistical analysis. No correlations were significant and thus specific conclusions regarding associations between training behaviors and injuries could not be made. The response rate was low, likely due to the fact the participants were asked to complete the surveys upon entering the tournament building. Athletes may have neglected to complete or return the surveys because of lack of time or feeling that it was not a priority prior to their match. Also, a self-report retrospective survey may be affected by poor recall and perception bias. For example, the recall of more severe and painful injuries would likely be better than that of minor injuries/trauma. The survey was also completed at a competition, thus those who were injured and not participating were already selected out. With respect to the information gained regarding weight cycling, actual weights were not taken. It would have been more informative to weigh the athletes at the mat just prior to their match and compare the result with that of their tournament weigh-in. As mentioned previously, the questionnaire used in this pilot study was vague regarding several concepts. Key definitions were not provided on the questionnaire. Future studies should ensure that all concepts are clearly defined in order to reduce subject confusion and hopefully avoid missing responses or poor response rates.

## Conclusion

The results of this pilot study are primarily descriptive. Even so, they highlight specific training habits and injuries among Taekwondo athletes. Although this pilot study examined a variety of pre-competition habits, it is evident that there are several specific areas which require more in-depth investigation. In order for safety recommendations to be implemented, it is likely that clear relationships will need to be demonstrated. Specifically, the physical effects of weight cycling on performance and improved training to avoid injury need to be examined. Athlete's perceptions and belief systems surrounding weight cycling, social support, and injury reporting are all topics in need of further investigation. As such, follow-up research on the relationship of pre-competition habits and injuries in Taekwondo athletes is necessary.

## Competing interests

The author(s) declare that they have no competing interests.

## Authors' contributions

MK analyzed the data, wrote the abstract and various components of the manuscript. HS wrote various components of the manuscript, as well as helped to edit it. YSC helped to develop, distribute and collect the questionnaire, and edit the manuscript.

## Pre-publication history

The pre-publication history for this paper can be accessed here:



## Supplementary Material

Additional File 1 There is also an additional file attached titled, "**Figure 1. Questionnaire**". This is the exact Questionnaire which was distributed to the athletes. Click here for file

## References

[B1] Buschbacher RM, Shay T (1999). Martial arts. Physical Medicine and Rehabilitative Clinics of North America.

[B2] Feehan M, Waller AE (1995). Precompetition injury and subsequent tournament performance in full-contact taekwondo. British Journal of Sports Medicine.

[B3] Pieter W, Zemper ED (1999). Head and neck injuries in young taekwondo athletes. Journal of sports medicine and physical fitness.

[B4] Koh JO, Watkinson EJ (2002). Possible concussions following head blows in the 2001 Canadian national taekwondo championships. Boundaries.

[B5] Zemper ED, Pieter W (1989). Injury rates during the 1988 US Olympic team trials for taekwondo. British Journal of Sports Medicine.

[B6] Kazemi M, Pieter W (2004). Injuries at a Canadian national taekwondo championship: a prospective study. BMC Musculoskeletal Disorder.

[B7] Website title. http://www.wtf.org.

[B8] (2005). Stedman's Medical Dictionary for the Health Professions and Nursing.

[B9] Fogelholm GM, Koskinen R, Laakso J, Rankinen T, Ruokonen I (1993). Gradual and rapid weight loss: Effects on nutrition and performance in male athletes. Medicine and Science in Sports and Exercise.

[B10] Hall CJ, Lane AM (2001). Effects of rapid weight loss on mood and performance among amateur boxers. British Journal of Sports Medicine.

[B11] Alderman BL, Landers DM, Carlson J, Scott JR (2004). Factors related to rapid weight loss practices among international-style wrestlers. Medicine & Science in Sports & Exercise.

[B12] Filare E, Maso F, Degoutte F, Jouanel P, Lac G (2001). Food restriction, performance, psychological state and lipid values in judo athletes. International Journal of Sports Medicine.

[B13] Worrell TW (1994). Factors associated with hamstring injuries: An approach to treatment and preventative measures. Sports Medicine.

[B14] Birrer RB (1996). Trauma epidemiology in the martial arts: The results of an eighteen-year international survey. American Journal of Sports Medicine.

[B15] Wenos DL, Amato HK (1998). Weight cycling alters muscular strength and endurance ratings of perceived exertion and total body water in college wrestlers. Perceptual and Motor Skills.

[B16] Smith M, Dyson R, Hale T, Hamilton M, Kelly J, Wellington P (2001). The effects of restricted energy and fluid intake on simulated amateur boxing performance. International Journal of Sport Nutrition and Exercise Metabolism.

[B17] Waslen PE, McCargar LJ, Taunton JE (1993). Weight cycling in competitive judokas. Clinical Journal of Sports Medicine.

[B18] Chapman C, Lane AM, Brierly JH, Terry PC (1997). Anxiety, self-confidence and performance in tae kwon-do. Perceptual and Motor Skills.

[B19] Finkenberg ME, DiNucci JM, McCune ED, McCune SL (1992). Analysis of the effect of competitive trait anxiety on performance in taekwondo competition. Perceptual and Motor Skills.

