# Supplementary material for: Pre-competition habits and injuries in Taekwondo athletes
Source: BMC Musculoskelet Disord. 2005 May 27;6:26. doi: 10.1186/1471-2474-6-26 (PMC1168901; doi:10.1186/1471-2474-6-26)
Supplement: Additional File 1 — There is also an additional file attached titled, "Figure 1. Questionnaire". This is the exact Questionnaire which was distributed to the athletes. [file 1471-2474-6-26-S1.doc]

# **Figure 1. Questionnaire**

# TAEKWONDO ATHLETE’S PROFILE

1. How many years have you been practicing Taekwondo?

a. 1 b. 2-3 c. 4-5 d. 6-7 e. 8+

2. How many times / week do you practice?

a. 2 b. 3 c. 4 d. 5-6 e. 7+

3. How many hours / session do you practice?

a. 1 b. 2 c. 3 d. 4 e. 5+

4. Are you satisfied with the frequency and length of your training?

YES NO

If NO, would you wish to increase or decrease the frequency of your training per week?

INCREASE DECREASE

If NO, would you like to increase or decrease the length of your training each session?

INCREASE DECREASE

5. How many times / week do you spar or practice sparring techniques?

a. 1-2 b. 3 c. 4 d. 5-6 e. 7+

6. How many hours / session do you spar or practice sparring techniques?

a. 1 b. 2 c. 3 d. 4 e. 5+

7. Generally, I stretch (choose one) training.

BEFORE AFTER BOTH

8. I do a warm-up other than stretching before kicking.

ALWAYS SOMETIMES NEVER

9. I do a cool-down other than stretching after training.

ALWAYS SOMETIMES NEVER

10. Do you wear protective gear when training?

ALWAYS SOMETIMES NEVER

11. If you do, which ones?

elbow pads shoes

shin pads gloves

Headgear instep pads

chest protector mouth guard

12. Do you fast before competition?

YES NO

13. If YES, what do you do?

a. Do not eat and drink and do aerobic exercise

b. Do not eat but drink and do aerobic exercise

c. Do not drink but eat and do aerobic exercise

14. Do you feel ready for the competition today?

a. YES b. NO

c. Yes, but nervous d. No, and nervous

15. If NO, what is the problem?

a. not enough b. coach c. parents d. peers e. personal

training

16. If COACH, what is the problem?

a. Coach does not like me b. I do not like the coach

c. Not enough directions d. Too much direction

e. Communication problem f. Cannot trust coach judgment

17. Are your parents supportive of your involvement in TKD?

YES NO Does not apply

18. Is your spouse or significant other supportive of your involvement in TKD?

YES NO Does not apply

19. What is your:

GENDER ______________ WEIGHT (lbs)_____________

# AGE ______________ HEIGHT (ft) _____________

20. I am:

Left-handed Right-handed

21. Injuries (see A through E following this chart for instructions on each category

you enter in the table):

|  | AInjury | **B**  **Type** | **C**  **# Practices**  **Missed** | **D**  **When** | **E**  **Professional**  **Attention** |
| --- | --- | --- | --- | --- | --- |
| e.g. | R/foot | contusion | 2 | TR | Physiotherapist |
| **A.** |  |  |  |  |  |
| **B.** |  |  |  |  |  |
| **C.** |  |  |  |  |  |
| **D.** |  |  |  |  |  |
| **E.** |  |  |  |  |  |

**A.** Which injuries (circle) are due to TKD this year: 1998?

#
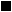
 Lower back
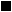
 head
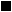
 mid-back
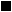
 neck


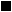
 Ribs R L
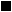
 hip R L

#
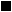
 Arm R L
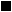
 Knee R L


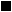
 Elbow R L
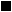
 Leg R L


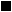
 Forearm R L
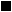
 Ankle R L


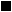
 Wrist R L
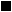
 Foot R L


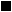
 Hand R L
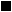
 OTHER (be specific)

**B.** What type of injury was it?


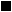
 Sprain/Strain
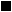
 Headache


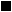
 Concussion
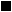
 Bruise/Contusion


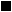
 Muscle Cramp
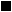
 Fracture


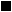
 OTHER (be specific)

**C.** How many practices did you miss as a result of this injury?

**D.** When did the injury occur?


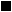
 Training: **TR**
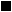
 Competition: **COMP**

**E.** Which professional did you see for this injury?


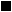
 Acupuncturist
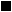
 Massage Therapist
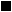
 Physiotherapist


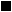
 Chiropractor
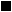
 Medical Doctor
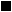
 None


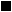
 OTHER (please specify)
